# Supplementary material for: Optical gating and streaking of free electrons with sub-optical cycle precision
Source: Nat Commun. 2017 Jan 25;8:14342. doi: 10.1038/ncomms14342 (PMC5288495; doi:10.1038/ncomms14342)
Supplement: Supplementary Information — Supplementary Figures, Supplementary Discussion and Supplementary References. [file ncomms14342-s1.pdf]

Supplementary figures

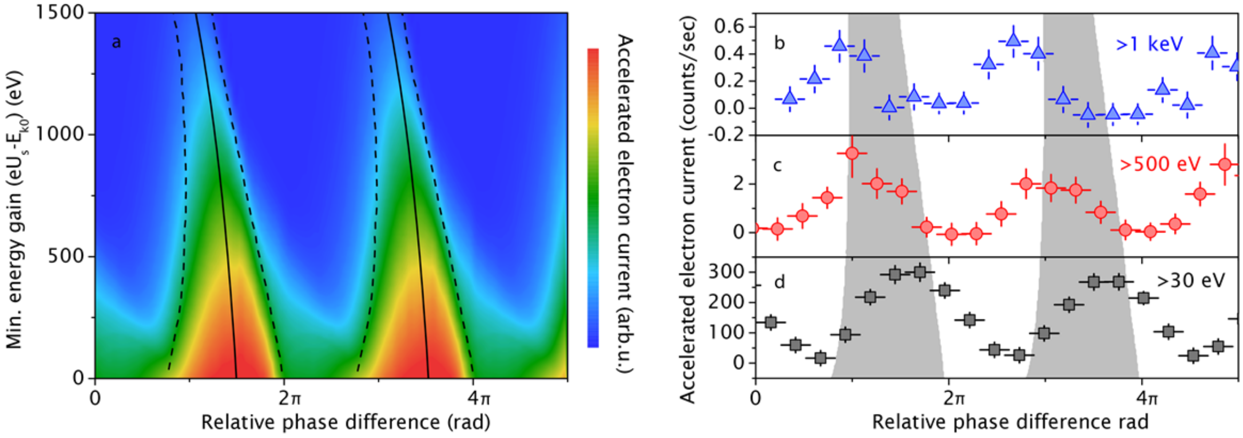

Supplementary Figure 1. Accelerated electron current as a function of minimum energy gain. a, Numerical simulation of the accelerated electron current (color scale) as a function of the relative phase difference between the two laser pulses and the minimum energy gain (eU_s-E_{k0}). Full curves indicate the phase values of the peak current. Dashed curves indicate the FWHM of the accelerated electron current peaks. **b**, **c**, **d** Measured phase dependent oscillations of the accelerated electron current for a minimum energy gain (eU_s-E_{k0}) of 30 eV (**d**, squares), 500 eV (**c**, circles) and 1 keV (**b**, triangles). The shaded areas shows the theoretical shift and narrowing of the peaks (full and dashed curves in **a**). The stronger phase shift observed in the experiment can be explained by higher-order phase components present in the laser pulses. However, these do not influence the gating and streaking performance of the presented scheme. Error bars in **b**, **c**, and **d** reflect the statistical error of individual measurements (accelerated electron current) and the timing stability of the experiment (relative phase difference).

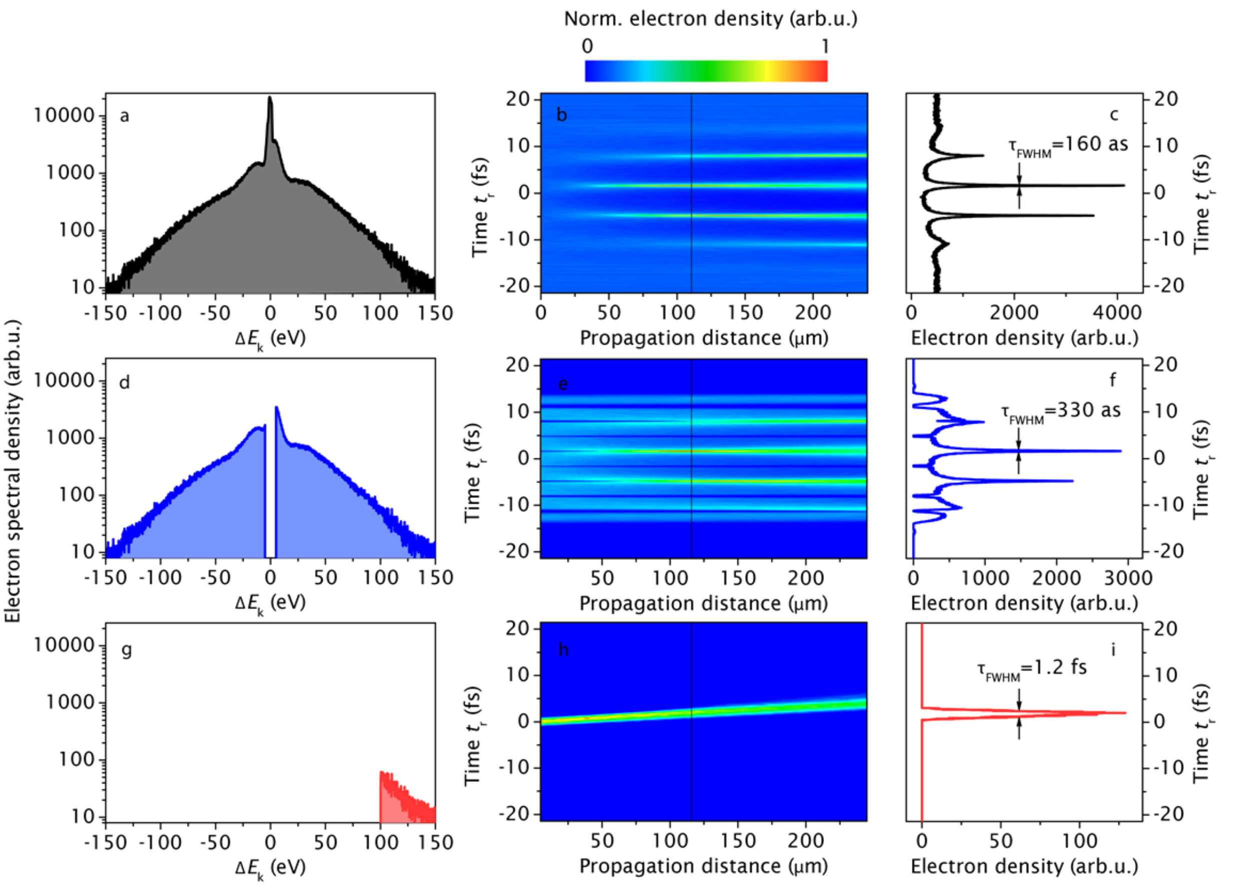

Supplementary Figure 2. High-pass energy filtering vs. attosecond microbunching. a, d, g, Different spectral parts of the electron beam after interaction with a few-cycle laser pulse. **a** Spectrum of the full beam, **d** a band-pass filtered spectrum, in which electrons within the initial energy distribution are filtered out, **g** the high-pass filtered spectrum proposed for single-cycle temporal gating of the electron beam. **b, e, h,** Spatio-temporal evolution of the density of electrons as a function of the retarded time $t_r=t-z/\beta_0 c$ and propagation distance z for electrons corresponding to the spectra shown in **a, d, g**. **c, f, i,** The temporal evolution of electron density post-interaction after freely propagating over a distance z=110 μm (indicated by black lines in **b, e, h**), where the maximal density for the full beam is reached. While the detection of electrons with spectra shown in **a, d** does not bring single cycle temporal resolution (only 18% of detected electrons are within the main attosecond bunch, the rest are distributed in a ~20 fs time window), high-pass filtering using the spectrum shown in **g** results in temporal gating of electrons within a temporal window of 1.2 fs.

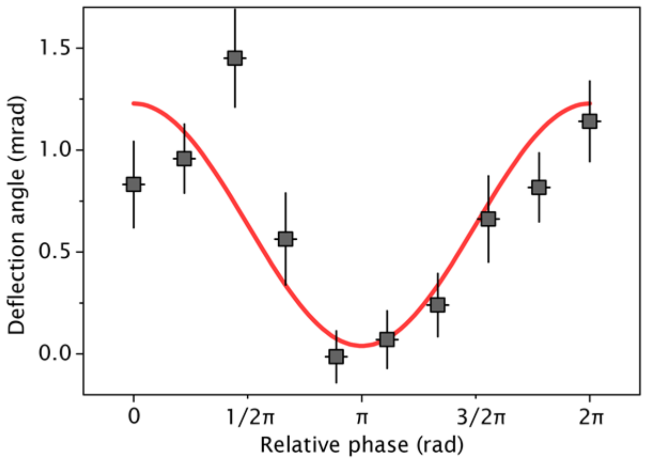

Supplementary Figure 3. Electron beam deflection as a function of relative phase between laser pulses. Measured deflection angle (squares) of the center of the accelerated electron beam with energy gain ΔE_k<30 eV (data shown in Figure 4) obtained by fitting the current distribution at each relative phase between the two laser pulses by error function compared to the expected sine dependence (red curve). Error bars of deflection angle were calculated from the statistical error of individual measurements and from the knife-edge positioning resolution while the error bars of relative phase correspond to timing stability of the experiment (200 as).

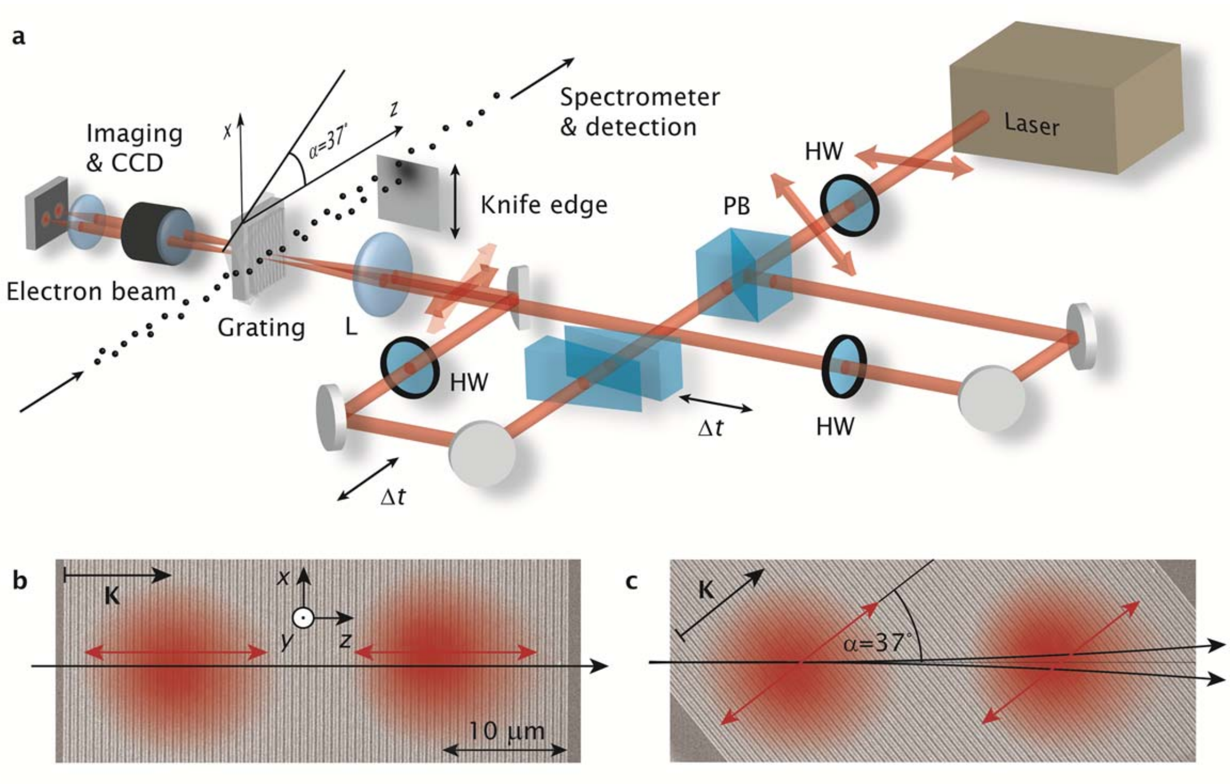

Supplementary Figure 4. Overview of the experimental setup. a, Experimental setup used for electron energy gating and streaking experiments with L being the focusing lens, PB the polarizing beamsplitter cube and HW the half-waveplates used for polarization adjustment. **b**, **c**, Scanning electron microscope images of the grating surface with the optical intensity of both laser pulses (red color) measured by the imaging system and a charge-coupled device camera used in the gating (**b**) and the streaking (**c**) experiments. Electron beam (black arrows), laser polarization (red arrows) and grating vector **K** are displayed.

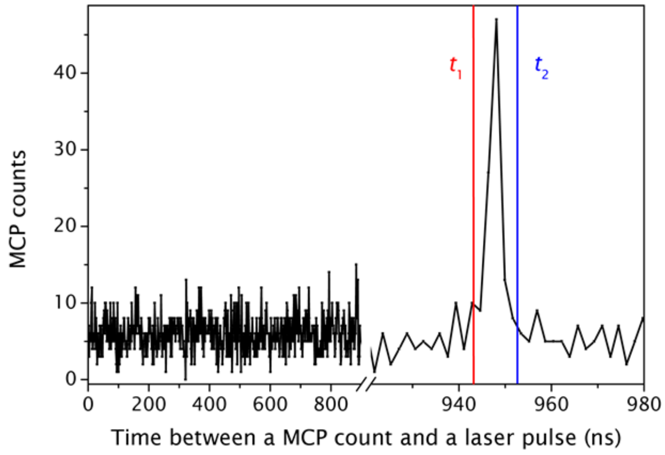

Supplementary Figure 5. Detected electron current. Histogram showing the number of counts on a micro-channel plate detector as a function of the delay between each micro-channel plate count and the following laser pulse detected by an avalanche photodiode (in the Materials and Methods referred to $S(t)$). The boundaries for the signal integration time window are labeled as t_1 and t_2. The average background count level per bin is calculated as $N_{\mathrm{bg}} = \int_0^{t_1} S(t)dt / t_1$.

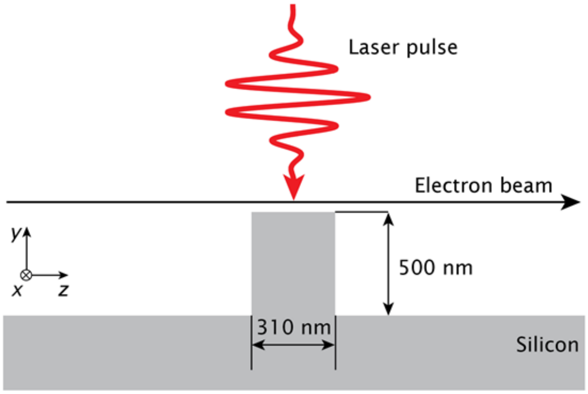

Supplementary Figure 6. Single-cycle gating operation. Side-view of the silicon nanostructure used in simulations of sub-optical cycle electron beam gating. The single grating tooth allows for the single-cycle temporal resolution shown in Figure 3.

Supplementary discussion

Experimental techniques for electron streaking by optical fields Energy modulation of the electron beam due to the interaction with femtosecond laser pulses has been demonstrated using different geometries, materials, as well as with various laser and electron beam parameters (see refs. [1-7]). Theoretical predictions of attosecond bunch formation after the interaction have been published [1,2], however, the temporal resolution for electron bunch characterization or temporal gating has been so far limited by the duration of the laser pulse envelope. Here we directly demonstrate the sub-optical-cycle energy structure of the electron beam in the time domain. This is possible due to the employed geometry, where the freely propagating electron beam interacts synchronously with subsequent optical cycles of the driving laser pulse modulated by the silicon grating. In contrast to previously published results, this scheme allows for straightforward experimental implementation of subsequent interactions with two or more laser pulses and thus allows for resolving sub-cycle electron dynamics.

Due to the synchronous interaction presented in this paper and the higher peak-fields that can be applied to transparent dielectric materials (GV/m range), a much higher energy gain can be achieved in comparison with competing schemes [1,3,5]. This allows for temporal gating of electron beams with broader initial energy distributions, with an energy width limited by the maximum achievable energy gain (1.3 keV in our experiments). While this is of smaller importance for TEM- or SEM-based schemes, this is of large interest for schemes operating with larger electron energies, such as MeV sources driven by RF-cavities [8]. Although the temporal resolution of ~1 fs was demonstrated recently using the RF deflection cavity [9], this approach suffers from the timing jitter of around 100 fs [10]. In contrast, using terahertz fields for electron control and metrology, timing stability better than 4 fs was achieved by all-optical timing control of the experiment [11] which can be improved to sub-100 as range when gating and streaking at optical frequencies is employed.

Our technique also offers close to 100% transmission of electrons. In contrast, e.g. the streaking technique presented in [3] is limited by electron transmission of the metallic film to ~3.5%. The main limitation of our scheme is given by the rapid sub-wavelength transverse spatial decay of the synchronous mode as discussed in Methods. However, this is an inherent property of the interaction of an electron beam with optical near-fields and thus applies also to other techniques based on this principle [1,5-7].

Electron beam emittance after interaction with optical near-fields

For applications of the temporal gating scheme in ultrafast electron microscopy and diffraction, transverse beam properties are ideally preserved. The geometrical properties of the beam can be described by the transverse emittance. Here we calculate the upper limit of the transverse emittance growth for electrons interacting with a laser pulse with a peak electric field of 1.3 GV/m (slightly below the laser damage threshold for the pulse duration and repetition rate of this work) with the laser beam and the electron beam parameters used in our experiments.

The RMS normalized transverse emittance in x-direction is defined as:

$$\varepsilon_x = \frac{1}{m_\mathrm{e} c}\sqrt{\langle x^2\rangle\langle p_x^2\rangle - \langle x p_x\rangle^2} \qquad (1)$$

where x is electron position in real space and p_x is the electron momentum in x-direction. In the case of the mode of the non-tilted grating (Supplementary Figure 4b), the force component in x-direction is zero and thus ε_x does not change after interaction. To obtain the emittance in the direction perpendicular to the grating surface (y), we integrate the number of 10^5 electrons arriving at the structure within one period of laser field (those which interact with the envelope-peak of the laser pulse). The emittance in this case changes from the initial value of $\varepsilon_y^{\mathrm{in}}$ =2.2×10^{-11} m.rad to the final value of $\varepsilon_y^{\mathrm{f}}$ =1.4×10^{-10} m.rad.

However, for temporal gating, only the emittance of electrons transmitted through the high-pass filter is relevant. For electrons with energy gain higher than 75% of maximum energy gain, the final emittance is only $\varepsilon_y^{\mathrm{f}}$ =3.7×10^{-11} m.rad.

High-pass energy gating vs. attosecond microbunching

In this section we compare the single-cycle temporal gating scheme proposed in this paper (calculations shown in Figure 3) with the attosecond ballistic velocity bunching which has been proposed previously. The latter is a consequence of the velocity modulation imprinted on electrons during interaction with optical near-fields [1] or with the ponderomotive potential of a moving intensity grating generated by interfering femtosecond laser pulses [2]. This ballistic velocity bunching also takes place in the case of the synchronous interaction presented in this paper. However, each attosecond bunch contains electrons with a broad energy spectrum centered around the initial beam energy (see Supplementary Figure 2a, the electron energy spectrum after interaction with a few-cycle laser pulse). It is thus extremely challenging to isolate electrons within a single attosecond bunch via spectral filtering unless a single-cycle laser pulse is used.

To demonstrate this, we show the spatio-temporal evolution of the electron density post-interaction for electrons from three different energy regions. The full beam (Supplementary Figure 2a, b, c, black spectrum) and band-pass filtered beam (electrons within the initial energy spectrum are filtered out, Supplementary Figure 2d, e, f, blue spectrum) both reveal attosecond bunch train formation. However, the temporal resolution in these two cases is not given by the single attosecond bunch duration (τ_{FWHM}=160 as and τ_{FWHM}=330 as, respectively) because the bunch with the highest peak density contains only 18 % of the electrons. The rest of the electrons are distributed within the time window given by the laser pulse envelope duration (~20 fs). In contrast to that, when high-pass energy filtering is applied (Supplementary Figure 2g, h, i, red spectrum), the electrons are temporally gated within a time window of 1.2 fs, limited by the fraction of the optical cycle of the driving laser pulse. The dynamics of electrons with different spectra is compared at the distance z=110 μm where the maximum electron density is reached (see

Supplementary Figure 2c, f, i). Calculations were performed via the theory described in Methods (laser wavelength λ=2 μm, pulse duration τ=10 fs, electron beam spot size w_e=70 nm, distance of the electron beam center from the structure surface d_e=100 nm).

Supplementary References

[1] Feist, A. *et al.* Quantum coherent optical phase modulation in an ultrafast transmission electron microscope. *Nature* **521**, 200-203 (2015).

[2] Baum, P. & Zewail, A. H. 4D attosecond imaging with free electrons: Diffraction methods and potential applications. *Chem. Phys.* **366**, 2–8 (2009).

[3] Kirchner, F. O., Gliserin, A., Krausz, F. & Baum, P. Laser streaking of free electrons at 25 keV. *Nat. Photonics* **8**, 52-57 (2014).

[4] Eckle, P. *et al*. Attosecond angular streaking. *Nat. Phys.* **4**, 565-570 (2008).

[5] Barwick, B., Flannigan, D. J. & Zewail, A. H. Photon-induced near-field electron microscopy. *Nature* **462**, 902-906 (2009).

[6] García de Abajo, F. J., Asenjo-Garcia, A. & Kociak, M. Multiphoton absorption and emission by interaction of swift electrons with evanescent light fields. *Nano Lett.* **10**, 1859–1863 (2010).

[7] Piazza, L. *et al.* Simultaneous observation of the quantization and the interference pattern of a plasmonic near-field. *Nat. Commun.* **6**, 6407 (2015).

[8] Hastings, J. B. *et al.* Ultrafast time-resolved electron diffraction with megavolt electron beams. *Appl. Phys. Lett.* **89**, 184109 (2006).

[9] Behrens, C. *et al.* Few-femtosecond time-resolved measurements of X-ray free-electron lasers. *Nat. Comm.* **5**, 3762 (2014).

[10] Röhrs, M. *et al.* Time-resolved electron beam phase space tomography at a soft x-ray free-electron laser. *Phys. Rev. Spec. Top.-Accel. Beams* **12**, 050704 (2009).

[11] Kealhofer, C. *et al.* All-optical control and metrology of electron pulses. *Science* **352**, 429-433 (2016).
