# Supplementary material for: Optical gating and streaking of free electrons with sub-optical cycle precision
Source: Nat Commun. 2017 Jan 25;8:14342. doi: 10.1038/ncomms14342 (PMC5288495; doi:10.1038/ncomms14342)
Supplement: Peer Review File [file ncomms14342-s2.pdf]

Transferred manuscripts:

Editorial Note: this manuscript has been previously reviewed at another journal that is not operating a transparent peer review scheme. This document only contains reviewer comments and rebuttal letters for versions considered at Nature Communications.
